# Antioxidant and Anti-Aging Effects of Porphyra-334 Produced from *Saccharomyces cerevisiae* in Human Skin Models

**DOI:** 10.3390/md24030098

**Published:** 2026-02-28

**Authors:** Soeun Park, Saitbyul Park, Nok Hyun Park, Eun-Soo Lee, Kilsun Myoung, Heung-Soo Baek, Jaewoo Jang, Sang-Jip Nam, Jaeyoung Ko, Chang Seok Lee

**Affiliations:** 1 Department of Senior Healthcare Major in Biopharmaceuticals and Major in Cosmetic Science, Eulji University, Seongnam-si 13153, Republic of Korea; thdms6642@gmail.com; 2 Advanced Beauty Science Research Division, AMOREPACIFIC Research & Innovation Center, Yongin-si 17074, Republic of Korea; sbpark0819@amorepacific.com (S.P.); aquareve@amorepacific.com (N.H.P.); soopian@amorepacific.com (E.-S.L.); ksmyoung@amorepacific.com (K.M.); monmimi@amorepacific.com (H.-S.B.); 3 CutisBio Research & Development Center, Seoul 06025, Republic of Korea; jwjang@cutisbio.com; 4 Department of Chemistry and Nanoscience, Ewha Womans University, Seoul 03760, Republic of Korea; sjnam@ewha.ac.kr

**Keywords:** porphyra-334, UV, human skin primary cells, human artificial skin tissue models, antioxidant, anti-aging

## Abstract

Porphyra-334 (PPR-334) is one of the species of mycosporine-like amino acids (MAAs), known as biological UV protection ingredients. In this study, we developed a large-scale purification process to extract PPR-334 from *Saccharomyces cerevisiae* and confirmed the previously identified efficacy of PPR-334, while also demonstrating its efficacy under UV-independent conditions. PPR-334 scavenged reactivity oxygen species (ROS) and increased catalase (CAT) gene expression in human epidermal keratinocyte cells (HEKa). In both HEKa and normal human dermal fibroblast cells (NHDF), PPR-334 suppressed the gene expression of matrix metalloproteinase-1 (MMP-1). NHDF treated with PPR-334 showed increased collagen expression and proliferation, while advanced glycation end-product (AGE) production was decreased. It was confirmed that the efficacy in vitro was also reproduced in human artificial skin tissue models. Above all, the antioxidant efficacy mechanism of PPR-334 through nuclear factor erythroid 2-related factor 2 (NRF2) and Caspase-9 signals was identified. It was determined that the proliferation efficacy of PPR-334 was due to factors related to the cell cycle. These results demonstrate the anti-aging efficacy of PPR-334 independent of UV irradiation, while enhancing the UV-blocking and antioxidant effects. Thus, we suggest the potential of PPR-334 as a sunscreen agent as well as a dual- or multifunctional material.

## 1. Introduction

Ultraviolet radiation (UV) causes various skin problems such as skin cancer and aging [1,2]. UVA (320–400 nm) can reach the epidermis and dermis, causing aging through matrix metalloproteinase-1 (MMP-1) expression, collagen degradation and advanced glycation end-product (AGE) production, especially in the dermis [3,4,5,6]. MMP-1 breaks down the structure of the dermal layer by unwinding and decomposing the triple helix of collagen [7,8]. AGEs are produced due to UVA exposure and oxidant stress [9]. AGEs bind to proteins in the extracellular matrix (ECM), deteriorating its function and disrupting its structure [10,11]. UVB (280–320 nm) mainly reaches the epidermal layer and induces reactive oxygen species (ROS) production and apoptosis due to oxidant stress [12,13]. ROS is an important factor in cell death and skin aging by inducing damage to lipids, proteins, and nucleic acids [14]. UVB also contributes to skin aging by inducing MMP-1 in keratinocytes [15,16]. The epidermis, as the outermost layer of the skin, serves as the primary protective barrier. Disruption of the epidermis can propagate more severe damage to the dermis and deeper layers. Consequently, protecting epidermal cells against UV and preserving the structural integrity of the dermis, which constitutes a major component of the skin, are considered pivotal strategies in the prevention of cutaneous aging [3,17,18].

Mycosporine-like amino acids (MAAs) are small, water-soluble secondary metabolites produced by a wide range of marine organisms. Characterized by a central cyclohexanone or cyclohexenimine ring, MAAs absorb ultraviolet (UV) radiation between 310 and 362 nm, thereby providing protection against UV-induced damage. Over evolutionary timescales, these compounds have emerged as a key photoprotective adaptation, enabling marine organisms to survive in environments exposed to intense solar radiation. Porphyra-334 (PPR-334) is one of the types of mycosporine-like amino acids (MAAs) and was first found in *Porphyra tenera* Kjellman [19]. MAAs are responsible for protecting organisms under UV irradiation by absorbing UVA and UVB radiation and dispersing the energy as heat [20]. There are about 30 species of MAAs, which differ in UV absorption capacity. The *Porphyra yezoensis* extract containing PPR-334 effectively prevented UV-induced DNA damage, suggesting the high UV protection efficacy of PPR-334 [21]. Additionally, PPR-334 has been spectroscopically characterized and shown to exhibit strong UVA absorption [22]. Due to these UV-blocking effects, PPR-334 has been studied to have antioxidant and anti-aging effects [23,24,25]. In addition to the efficacy of UV protection, studies have been conducted in various ways to demonstrate that MAAs directly scavenge ROS [20,26].

**Figure 1:**
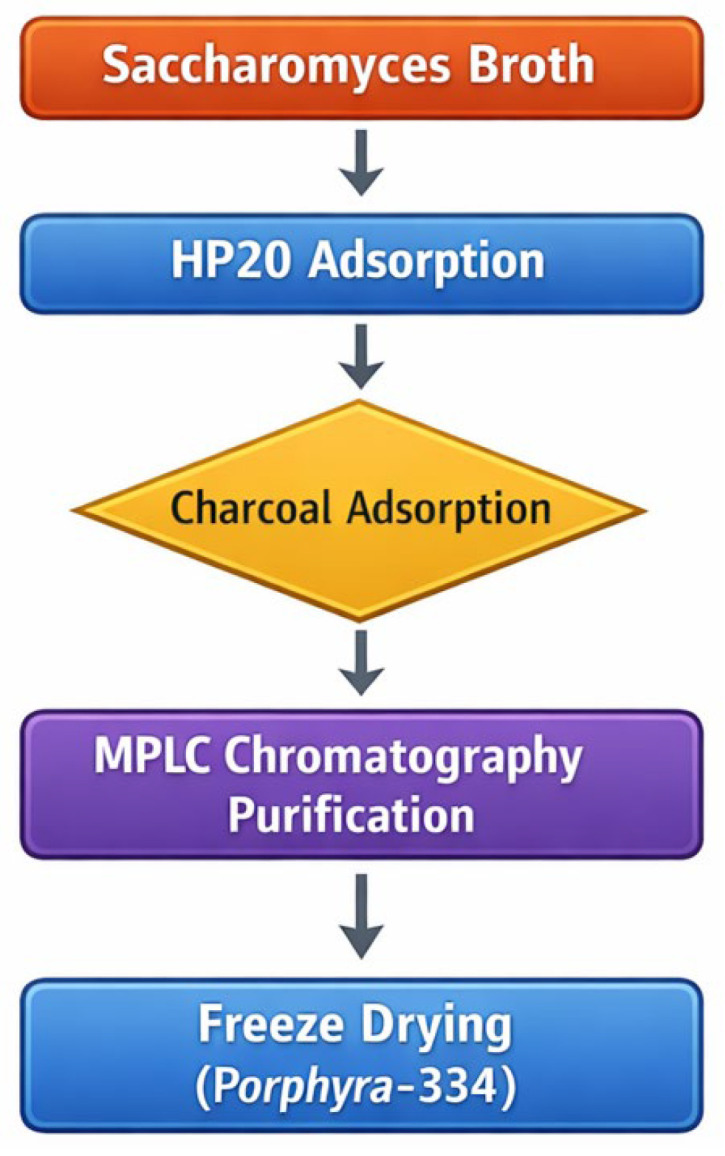
Schematic overview of the engineered yeast-based production and purification workflow of PPR-334.

MAAs are scarce in nature and their high aqueous polarity together with their structural similarity makes purification particularly challenging [27], leading to low overall yields and persistent obstacles that restrict industrial application. Laver (*Porphyra*/*Pyropia* spp.) is known to naturally contain high levels of mycosporine-like amino acids (MAAs), among which porphyra-334 is one of the major components. Previous studies have reported that porphyra-334 is present in *Porphyra* species at the level of several hundred micrograms to a few milligrams per gram of dry biomass, depending on species and environmental conditions [28,29]. Recently, Kim et al. reported the successful large-scale biosynthesis of Porphyra-334 in engineered *Saccharomyces cerevisiae*, establishing yeast as a promising microbial platform for sustainable production of MAAs [30]. In the present study, we utilized the same engineered yeast strain as reported by Kim et al. as the production host and further advanced this platform by developing a practical and scalable purification process for Porphyra-334. We also developed an integrated purification strategy consisting of solid-phase extraction followed by liquid chromatography, enabling efficient isolation of the compound from both cell biomass and culture medium and providing a scalable source for cosmetic applications. A schematic overview of the entire production and purification process is provided in Figure 1.

As such, research on MAAs has been conducted in various ways from a biological, chemical, and physical contexts. However, since the efficacy of MAAs in vitro has been mainly confirmed using cell lines, and its efficacy has also been evaluated under UV irradiation, little has been done for its independent effect. In addition, the number of studies focused solely on PPR-334 was significantly smaller than that of total MAAs. Therefore, in this study, we aimed to verify the previously reported efficacy of PPR-334 using primary cells and human artificial skin tissue models, and furthermore, to establish its potential as a functional ingredient by identifying its efficacy independent of UV irradiation.

## 2. Results

### 2.1. PPR-334 Exhibits Potent Radical Scavenging Activity and Enhanced Catalase (CAT) Expression

**Figure 2:**
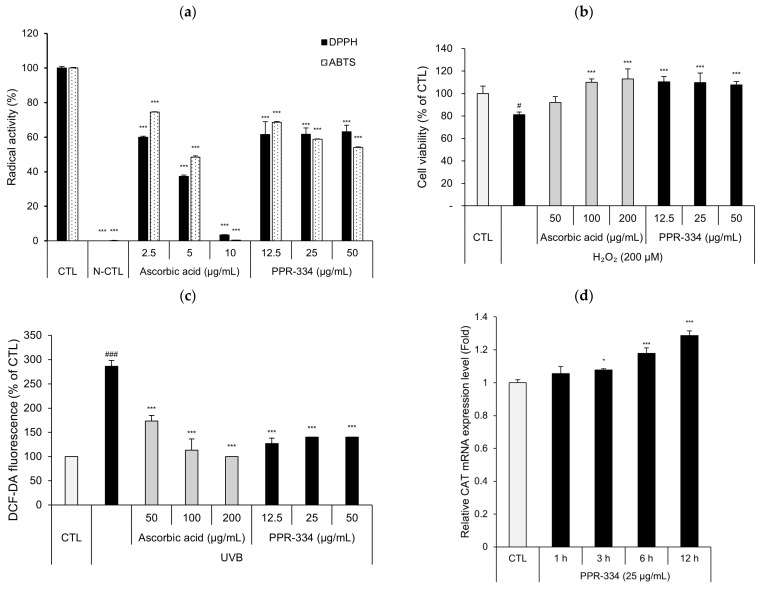
Antioxidant effects of PPR-334. (**a**) DPPH radical and ABTS radical activity of ascorbic acid (positive control group) and PPR-334. (**b**) Cell viability recovery effects of ascorbic acid (positive control group) and PPR-334 on H_2_O_2_-induced cytotoxicity in HEKa. HEKa treated for 24 h. (**c**) HEKa were treated with ascorbic acid (positive control group) and PPR-334 for 3 h, followed by DCF-DA staining and UVB exposure. Quantification of ROS levels was measured by DCF-DA fluorescence. (**d**) HEKa were treated with PPR-334 (25 µg/mL) for 1 h, 3 h, 6 h and 12 h. The gene expression level of CAT was measured using RT-qPCR. Results are presented as mean ± SD. (**a**) *** *p* < 0.001 vs. respective control group (DPPH and ABTS, respectively). (**b**) # *p* < 0.05 vs. control group. *** *p* < 0.001 vs. H_2_O_2_ alone. (**c**) ### *p* < 0.001 vs. control group. *** *p* < 0.001 vs. UVB alone. (**d**) * *p* < 005, *** *p* < 0.001 vs. control group.

To assess the radical scavenging activity of PPR-334 (Supplementary Information), 2,2-diphenyl-1-picrylhydrazyl (DPPH) and 2,2′-azino-bis(3-ethylbenzothiazoline-6-sulfonic acid) (ABTS^+^•) scavenging assays were performed. In addition, to evaluate the radical scavenging activity of PPR-334 in human skin cells, we used human epidermal keratinocyte cells (HEKa) and normal human dermal fibroblast cells (NHDF). H_2_O_2_-induced cell death inhibition assays were performed in both HEKa and NHDF at stable concentrations determined by cytotoxicity assay (Figures S1 and S2). 2′,7′-dichlorofluorescin diacetate (DCF-DA) assays were conducted in HEKa to evaluate intracellular ROS levels. The mRNA expression of catalase (CAT) in PPR-334 treated HEKa was measured by qRT-PCR at different time points to evaluate antioxidant-related enzyme expression. The results of DPPH and ABTS^+^• scavenging assays demonstrated that PPR-334 (12.5–50 µg/mL) exhibited antioxidant activity comparable to 5 µg/mL of ascorbic acid (Figure 2a, Table S1). In the H_2_O_2_-induced cell death inhibition assay, treatment with PPR-334 markedly reduced cell death, which was induced to approximately 20–30% by H_2_O_2_ (Figure 2b and Figure S3). Also, in DCF-DA assays, it was confirmed that the levels of ROS increased by UVB exposure were decreased by the treatment of PPR-334 (Figure 2c). The DCF-DA fluorescence inhibition rate of 50 µg/mL of ascorbic acid was approximately 61%, but the inhibition rate of PPR-334 (12.5–50 µg/mL) was about 86%, exceeding that of ascorbic acid (Table S2). The gene expression level of CAT increased from 3 h after PPR-334 treatment, peaking at 12 h (Figure 2d). Furthermore, the gene expression of CAT was also increased in NHDF treated with PPR-334 for 24 h (Figure S4). These findings suggest that PPR-334 has a ROS-scavenging effect and upregulates antioxidant-related enzyme in human skin cells.

### 2.2. Anti-Aging Effect of PPR-334 via MMP-1 Expression and Collagen Upregulation

**Figure 3:**
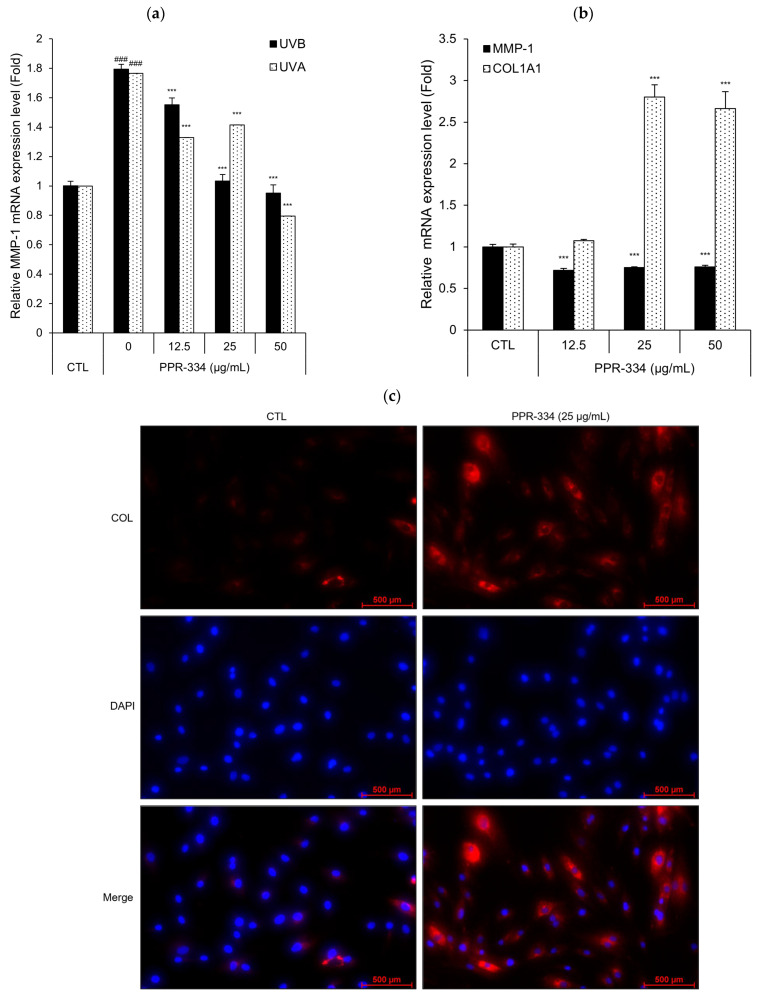

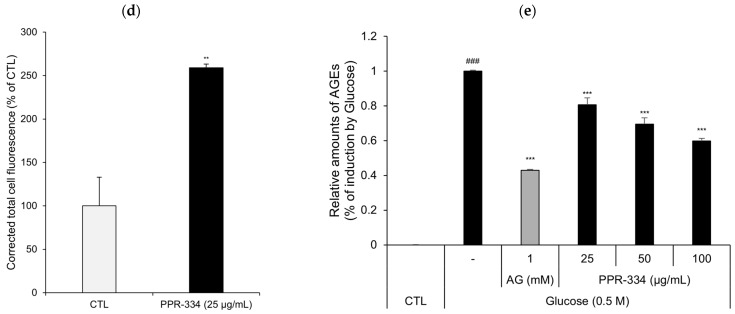
Anti-aging effects of PPR-334 in human skin cells. (**a**) HEKa were exposed to UVA (or UVB) in the presence of PPR-334. After UV-exposed, HEKa were treated with PPR-334 for 6 h. The gene expressions of MMP-1 were measured using RT-qPCR. (**b**) NHDF were treated with PPR-334 for 24 h. The gene expressions MMP-1 and COL1A1 were measured using RT-qPCR. (**c**,**d**) Immunocytochemistry of collagen I in NHDF treated with PPR-334 (25 µg/mL) for 24 h (scale bar = 620 µm). (**e**) Formation of AGEs was measured using cell-free glycation assay under treatment with glucose and PPR-334. AG served as a positive control. Results are presented as mean ± SD. (**a**) ### *p* < 0.001 respective control groups (UVB and UVA, respectively). *** *p* < 0.001 vs. respective UVB or UVA alone. (**b**) *** *p* < 0.001 vs. respective control groups (MMP-1 and COL1A1, respectively). (**d**) ** *p* < 0.01 vs. control group. (**e**) ### *p* < 0.001 vs. control group. *** *p* < 0.001 vs. glucose alone.

The anti-aging effect of PPR-334 was evaluated thorough analysis of the MMP-1 mRNA expression level and collagen expression in HEKa and NHDF. To assess its efficacy in the ultraviolet condition environment in keratinocytes, UVA or UVB were exposed, respectively, and the levels of MMP-1 gene expression were measured. To confirm the efficacy of PPR-334 alone in dermal cells, the mRNA expression levels of MMP-1 and collagen type I alpha 1 (COL1A1) were measured at 24 h after PPR-334 treatment. ICC was performed to visually confirm intracellular collagen I expression. In HEKa, PPR-334 treatment downregulated the MMP-1 mRNA expression, which induced by UVA/B exposure (Figure 3a). In NHDF, the MMP-1 mRNA expression was reduced by PPR-334, whereas the COL1A1 mRNA expression was significantly increased (Figure 3b). Immunocytochemistry (ICC) analysis confirmed the collagen-upregulating effect of PPR-334 (Figure 3c). The collagen expression level of the PPR-334 treatment group increased by more than twofold compared with the control group (Figure 3d). Compared with the positive control aminoguanidine, PPR-334 significantly inhibited the formation of AGEs in the cell-free glycation model. The inhibitory effect was dose-dependent across the tested range (25–100 µg/mL) (Figure 3e). Consequently, these findings demonstrate that PPR-334 has an anti-aging effect by regulating MMP-1 and collagen expression and suppressing glycosylation.

### 2.3. PPR-334 Promotes Proliferation and Increases Growth Factors Expression in Human Skin Cells

**Figure 4:**
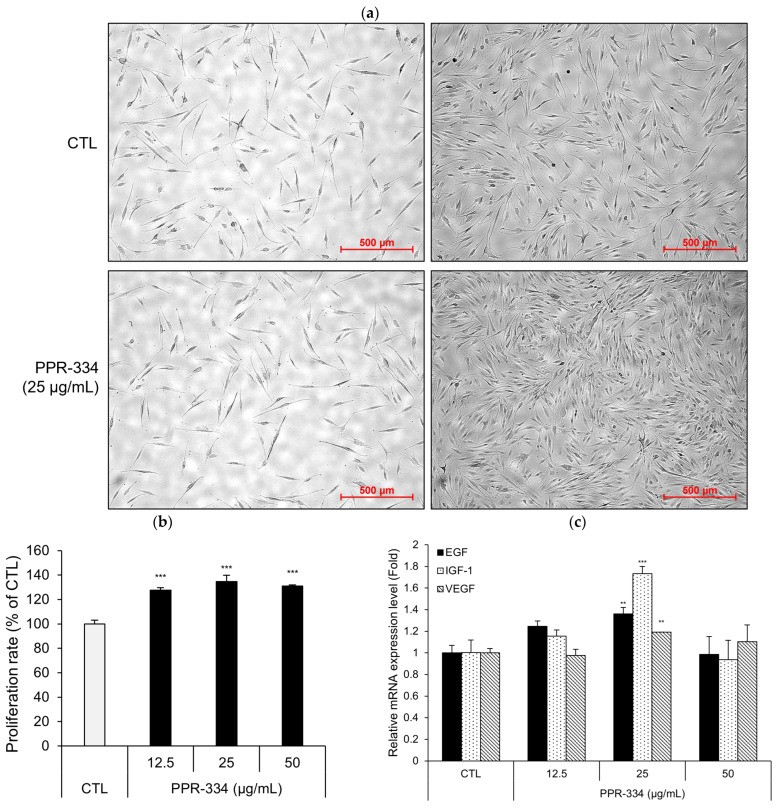
Proliferation effect of PPR-334 in human fibroblast. (**a**) NHDF were treated with PPR-334 (25 µg/mL) and monitored using live cell imaging system up to 72 h (scale bar = 500 µm). (**b**) NHDF were treated with PPR-334 for 72 h and determined using WST-8 assay. (**c**) NHDF treated with PPR-334 for 24 h. The gene expressions of EGF, IGF-1 and VEGF were measured using RT-qPCR. Results are presented as mean ± SD. (**b**) *** *p* < 0.001 vs. control group. (**c**) ** *p* < 0.01, *** *p* < 0.001 vs. respective control groups (EGF, IGF-1 and VEGF, respectively).

To investigate the proliferative effect of PPR-334 in NHDF, we used a live-cell imaging system. Then, the cell proliferation rate was quantified using a WST-8 assay. In addition, the mRNA expression levels of epidermal growth factor (EGF), insulin-like growth factor-1 (IGF-1), vascular endothelial growth factor (VEGF)—growth factors associated with proliferation—were measured. Visually, we can recognize the proliferation effect of PPR-334 (Figure 4a). The proliferation rate measured by WST-8 assay showed that PPR-334 treatment increased NHDF proliferation by approximately 30% (Figure 4b). The mRNA expression levels of EGF, IGF-1, and VEGF were elevated by PPR-334 treatment and show the highest levels observed at 25 µg/mL of PPR-334 (Figure 4c). In addition, PPR-334 treatment also increased cell proliferation in HEKa (Figure S5). Collectively, these results indicate that PPR-334 promotes proliferation in human skin cells.

### 2.4. Comprehensive Evaluation of PPR-334 Efficacy on Human Artificial Skin Tissue Models

**Figure 5:**
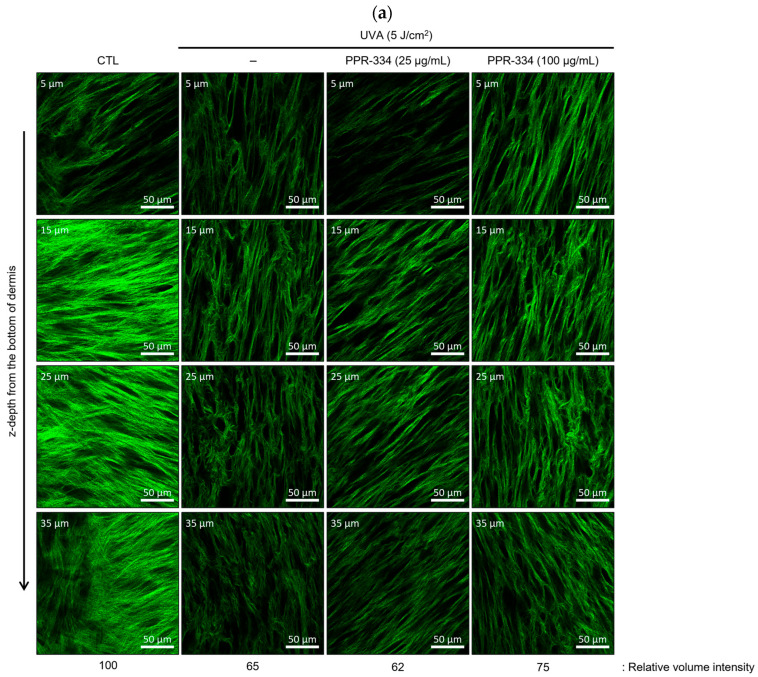

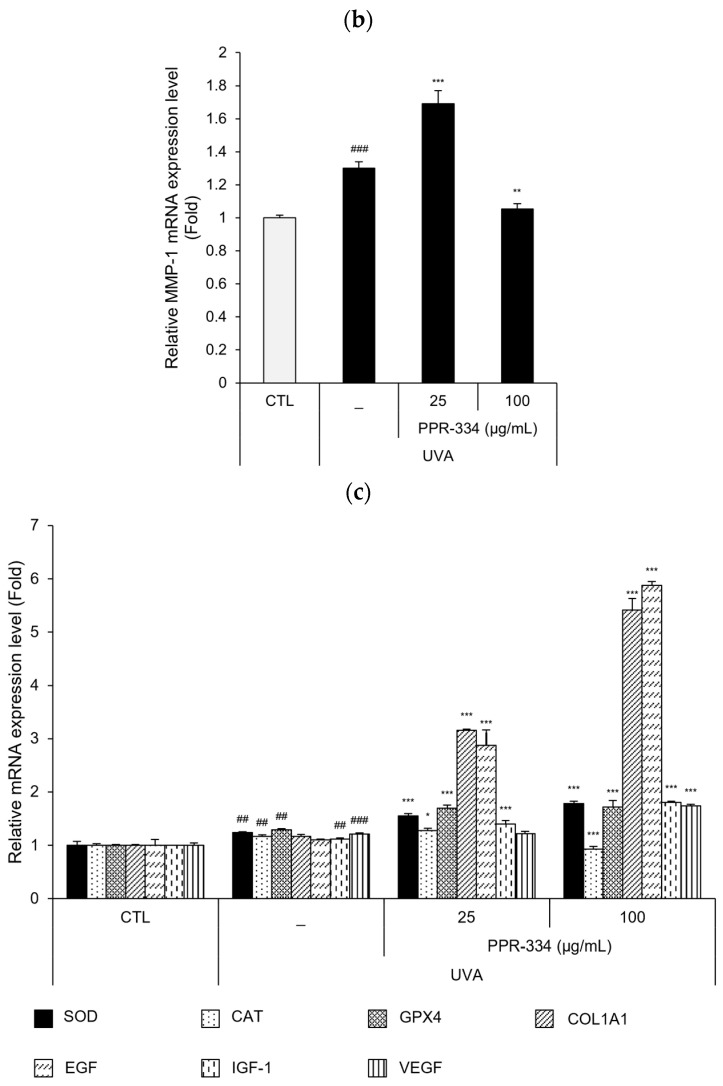
Validation of in vitro efficacy of PPR-334 human artificial skin tissue models. The skin equivalent was co-treated UVA (5 J/cm^2^) and PPR-334 and incubated for 48 h. (**a**) Multiphoton microscopic images of collagen fibers according to z-depth in skin equivalent (scale bar = 20 µm). (**b**) The gene expression of MMP-1 was measured using RT-qPCR. (**c**) The gene expressions of SOD, CAT, GPX4, COL1A1, EGF, IGF-1 and VEGF were measured using RT-qPCR. Results are presented as mean ± SD. (**b**) ### *p* < 0.001 vs. control group. ** *p* < 0.01, *** *p* < 0.001 vs. UVA alone. (**c**) ## *p* < 0.01, ### *p* < 0.001 vs. control group. * *p* < 0.05, *** *p* < 0.001 vs. respective UVA alone groups (SOD, CAT, GPX4, COL1A1, EGF, IGF-1 and VEGF, respectively).

We used a full-thickness human skin equivalent to investigate whether PPR-334 has an anti-aging effect to recover the disrupted collagen fivers in UVA-irradiated 3D environment. Under UVA-irradiation conditions, treatment of damaged skin equivalent with PPR-334 at 100 μg/mL for 48 h attenuated collagen fiber degradation. This protective effect against UVA-induced damage (both intensity and density of collagen fibers) was confirmed by multiphoton microscopy using a second harmonic generation technique (Figure 5a). The mRNA expression levels of MMP-1, superoxide dismutase (SOD), CAT, glutathione peroxidase 4 (GPX4), COL1A1, EGF, IGF-1, and VEGF in the human artificial skin tissue models were measured using RT-qPCR assay. Under UVA irradiation, 25 μg/mL of PPR-334 had no effect on MMP-1 reduction, while the MMP-1 mRNA expression levels were suppressed at 100 μg/mL of PPR-334 (Figure 5b). Although UVA stimulation did not affect the expression of antioxidant enzymes, collagen, and growth factors, it was confirmed that all factors were increased by treatment of PPR-334 (Figure 5c). These findings suggest that the in vitro efficacy of PPR-334 was further confirmed through comprehensive evaluation on human artificial skin tissue models.

### 2.5. Mechanism of Antioxidant Effect of PPR-334 in HEKa

**Figure 6:**
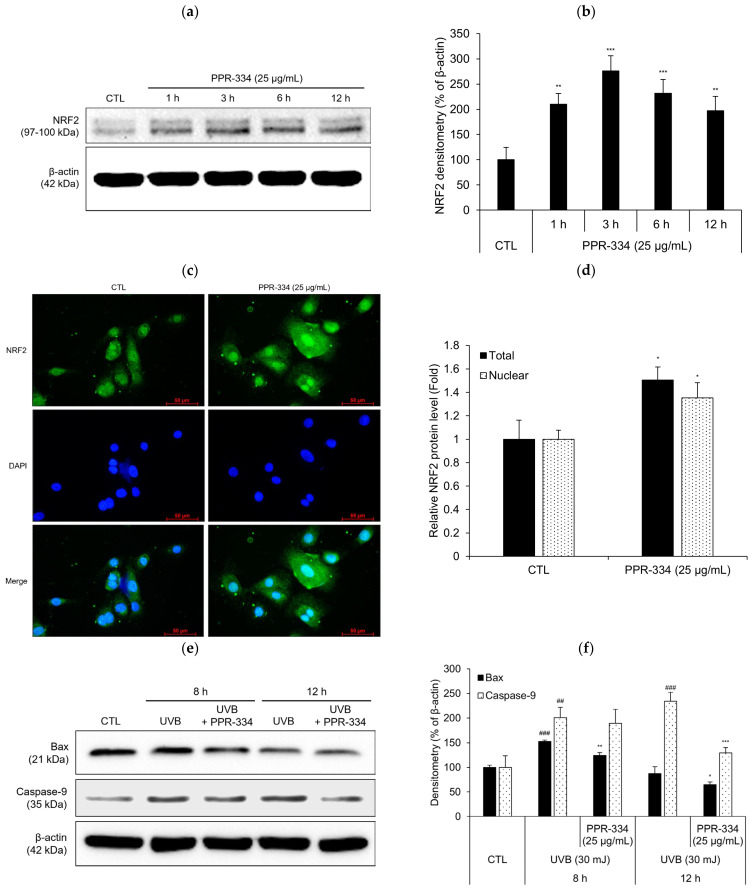
Proposed mechanisms of antioxidant effects of PPR-334 in HEKa. (**a**,**b**) HEKa were treated with PPR-334 (25 µg/mL) for 1 h, 3 h, 6 h and 12 h. The protein level of NRF2 was discovered by Westen blot assay. (**c**,**d**) Immunocytochemistry of NRF2 in HEKa treated with PPR-334 (25 µg/mL) for 3 h (scale bar = 50 µm). (**e**,**f**) HEKa were exposed to UVB (30 mJ/cm^2^) in the presence of PPR-334 (25 µg/mL) and then treated with PPR-334 (25 µg/mL) for 8 h and 12 h. The protein levels of Bax and Capase-9 were discovered by Western blot assay. Protein expression levels were quantified by densitometric analysis and normalized to β-actin. Results are presented as mean ± SD. (**b**) ** *p* < 0.01, *** *p* < 0.001 vs. control group. (**d**) * *p* < 0.05 vs. control group. (**f**) ## *p* < 0.01, ### *p* < 0.001 vs. control group. * *p* < 0.05, ** *p* < 0.01, *** *p* < 0.001 vs. UVB alone.

To investigate the mechanism of antioxidant efficacy of PPR-334 alone in HEKa, nuclear factor erythroid 2-related factor 2 (NRF2) expression was measured using Western blot analysis at 1–12 h after PPR-334 (25 µg/mL) treatment (Figure 6a). NRF2 expression was increased by PPR-334 treatment and the expression of the 3 h treatment group was the highest (Figure 6b). In addition, ICC analysis revealed an approximately 1.5-fold increase in total NRF2 levels and a 1.3-fold increase in nuclear NRF2 levels (Figure 6c,d).

Whereas the antioxidant mechanism was examined under PPR-334 single-treatment conditions, to assess the mechanism underlying cell survival effect via ROS scavenging, Caspase-9 and Bcl-2-associated X protein (Bax) expression was measured at 8 h and 12 h after co-treatment with UVB (30 mJ/cm^2^) and PPR-334 (25 µg/mL). In the results, Bax was weakly but significantly inhibited at 8 h after PPR-334 treatment, and Caspase-9 was inhibited at 12 h after PPR-334 treatment in UVB-irradiated HEKa (Figure 6e,f). These results suggest that PPR-334 enhances the intrinsic antioxidant defense capacity of keratinocytes through NRF2 activation under stimulus-free conditions, while concurrently attenuating apoptotic signaling under oxidative stress conditions.

### 2.6. Mechanism of Proliferation Effect of PPR-334 in NHDF

**Figure 7:**
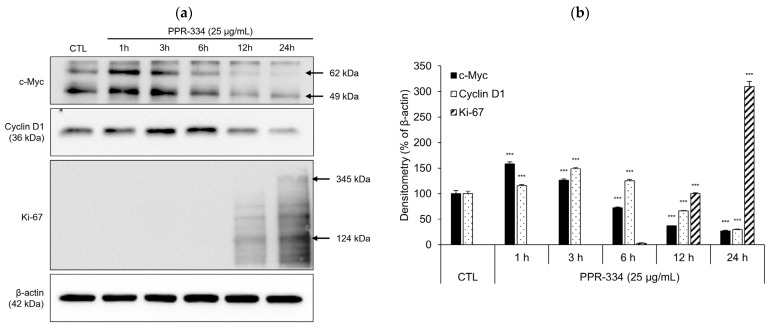
Proposed mechanisms of proliferation effects of PPR-334 in NHDF. (**a**,**b**) NHDF were treated with PPR-334 (25 µg/mL) for 1 h, 2 h, 3 h, 6 h, 12 h and 24 h. The protein levels of c-Myc, Cyclin D1 and Ki-67 were discovered by Western blot assay. The images shown are representative of three independent experiments. Protein expression levels were quantified by densitometric analysis and normalized to β-actin. Results are presented as mean ± SD. (**b**) *** *p* < 0.001 vs. control group.

In NHDF, to elucidate the proliferation-promoting mechanism of PPR-334, we examined the expression of cellular myelocytomatosis oncogene (c-Myc), G1/S-specific cyclin D1 (Cyclin D1), and marker of proliferation Ki-67 (Ki-67)—factors related to the cell cycle. The expression of c-Myc was increased at 1 h and 3 h after treating with PPR-334, and Cyclin D1 expression was maximized at 3 h and 6 h. After more than 12 h of PPR-334 treatment, Ki-67 was detected (Figure 7a,b). Notably, the expression pattern of these markers suggests a sequential activation of cell cycle progression, with early induction of c-Myc followed by Cyclin D1 upregulation and subsequent appearance of Ki-67 at a later time point, indicating that PPR-334 promotes fibroblast proliferation through activation of a cell cycle-related regulatory pathway.

## 3. Discussion

Porphyra-334 (PPR-334), a representative mycosporine-like amino acid (MAA), has recently gained attention as a promising natural sunscreen agent due to its excellent UV-absorbing and photoprotective properties. Preliminary small-scale clinical evaluations have begun to explore its UV-blocking and anti-aging effects, while research into scalable production methods for industrial applications is actively underway [30,31].

MAAs such as PPR-334 are water-soluble compounds derived from natural sources and are considered safe for cosmetic use. However, their high solubility in water has posed significant challenges for purification, traditionally requiring small-scale HPLC systems that are not suitable for mass production. Moreover, the natural abundance of PPR-334 in marine algae and microalgae is extremely low, making large-scale extraction impractical and inefficient. To overcome these limitations, we employed a synthetic biology approach to biosynthesize PPR-334 using engineered *Saccharomyces cerevisiae*, enabling high-yield production independent of natural sources. In this study, we developed a large-scale purification process using a preparative column-based method, allowing efficient isolation of high-purity PPR-334 from the biosynthetic mixture. The purified compound demonstrated excellent efficacy, and the newly established process enables its direct incorporation into cosmetic formulations.

As reported in the previous study [25], we confirmed the antioxidant efficacy of PPR-334 in the current study. In addition to reducing ROS levels, as confirmed by DCF-DA assay, it was indicated that PPR-334 exerts antioxidant effects independent of its UV-absorbing properties. In this study, we demonstrated that PPR-334 attenuates H_2_O_2_-induced cell death, accompanied by an increased expression of CAT, a key antioxidant enzyme involved in H_2_O_2_ detoxification, under PPR-334 treatment alone. In addition, under UVA-stimulated conditions in a 3D skin model, PPR-334 treatment resulted in increased expression of not only CAT but also SOD and GPX4. However, the present study did not comprehensively assess changes in antioxidant enzymes other than CAT under PPR-334 treatment alone, which represents a limitation. Further studies evaluating additional antioxidant enzymes, such as SOD and GPX, will be required to more clearly elucidate the intrinsic antioxidant effects of PPR-334. Nevertheless, we obtained that the expression of NRF2, a key regulator of the cellular antioxidant defense system, was significantly increased by PPR-334 treatment alone. In a study by Gacesa et al., it was reported that PPR-334 exhibits antioxidant efficacy via the Kelch-like ECH-associated protein 1 (Keap1)-NRF2 signaling pathway for oxidative stress caused by UVA exposure in a human fibroblast cell line [32]. In Figure 6a of this study, we observed that NRF2 expression increased as early as 1 h after PPR-334 treatment and remained elevated up to 12 h. This sustained upregulation may indicate a positive-feedback mechanism following NRF2 nuclear translocation. Consistent with this notion, ICC analysis demonstrated increased nuclear NRF2 fluorescence upon PPR-334 treatment (Figure 6c), suggesting that PPR-334 increased not only intracellular NRF2 expression but also its accumulation in the nucleus, which is associated with NRF2 activation and subsequent antioxidant response.

In addition, PPR-334 attenuated the UVB-induced upregulation of Bax and Caspase-9, indicating a mitigation of mitochondrial-dependent oxidative stress. Bax expression was significantly suppressed at 8 h after PPR-334 treatment, and Caspase-9 expression was significantly suppressed at 12 h after PPR-334 treatment. These results suggest that PPR-334 has potential anti-apoptotic activity by suppressing the expression of Caspase-9, which is involved in UVB-induced apoptosis, and the activation of Bax, an upstream apoptosis regulator [33,34]. Although, for PPR-334 to demonstrate its anti-apoptotic efficacy, additional verification of other factors, such as Bcl-2, Apaf-1 and p53 expression, is necessary, based on the results of this study, we believe that the anti-apoptotic effect of PPR-334 is evident under UVB irradiation conditions and that this may be related to its antioxidant activity.

According to a study published by Ryu et al., PPR-334 inhibited UVA-induced increased MMP-1 expression and ECM component destruction in human fibroblasts [35]. Studies on the efficacy of MAA alone, including PPR-334, are generally scarce. In a study conducted by Kim et al., PPR-334 alone treatment enhanced collagen expression in human Detroit 551 fibroblast cells, but the results were obtained using relatively low concentrations of PPR-334 [31]. We confirmed that PPR-334 inhibited UVA/B-induced expression of MMP-1 in HEKa, and treatment with a high concentration of PPR-334 alone in NHDF showed decreased MMP-1 gene expression and increased collagen expression. Furthermore, we demonstrated a proliferation effect in NHDF and anti-glycation effect of PPR-334. While studies have investigated the wound healing and regeneration effect of PPR-334 [36,37], its anti-aging potential—particularly regarding proliferation efficacy and the mechanisms in human dermal cells—has not been sufficiently explored. According to a study conducted by Waditee-Siriattha et al., MAAs including PPR-334 may exhibit anti-glycation activity in vitro, whereas a clear reduction in AGEs was not observed in animal models [38]. In the present study, we employed a cell-free anti-glycation assay to evaluate the potential suppression of AGE formation at the molecular level, and the results suggest that the anti-glycation potential of PPR-334 may vary depending on the type of stimulus applied. Through this study, we supported the anti-aging effect by newly elucidating the proliferation of dermal cells through cell cycle regulation of PPR-334. Taken together, this study demonstrates the anti-aging efficacy of PPR-334 through a reduction in MMP-1 gene expression, the enhancement of collagen levels, and the promotion of proliferation in NHDF. Although these findings suggest that the anti-aging effect is largely attributable to transcriptional regulation of MMP-1, the possibility of tissue inhibitors of metalloproteinase (TIMPs)-mediated inhibition of MMP-1 activity cannot be completely excluded [39,40]. Therefore, future mechanistic studies will be necessary to evaluate the potential contribution of TIMPs to this regulatory process.

In addition to these findings, our study also presents several important distinctions from previous reports of PPR-334. First, we demonstrated that PPR-334 exhibits intrinsic antioxidant and anti-aging effect even in the absence of UV exposure, indicating biological functions beyond its well-known UV-absorbing capacity. Second, the UV-protective and photoaging-related effects of PPR-334 previously reported in the literature [21,22,23,25] were reduced in a human full-thickness skin equivalent model, while its intrinsic, stimulus-independent effects were newly demonstrated in human primary skin cells. This approach provides a more physiologically relevant evaluation compared with studies relying solely on immortalized cell lines [23,24]. Third, we utilized a highly purified PPR-334 obtained through a controlled biosynthetic and preparative-scale purification process, allowing consistent evaluation and supporting its feasibility for scalable industrial applications. Collectively, these characteristics distinguish our work from earlier studies and broaden the current understanding of PPR-334 as a multifunctional skin-beneficial compound.

NRF2 is known to be involved in anti-inflammatory properties through the Keap1 and Nuclear Factor kappa B (NF-_K_B) pathways as well as antioxidant [41,42]. Studies on the anti-inflammatory efficacy through the NRF2-NF-_K_B pathway in PPR-334 have also been conducted [35]. Although this study did not address anti-inflammatory activity, the NRF2 upregulation observed with PPR-334 raises the possibility of such an effect and suggests that its range of efficacy could extend beyond the outcomes evaluated here.

In this study, we proposed the proliferation efficacy and mechanisms of dermal cells under PPR-334 in terms of anti-aging. EGF and IGF are closely related to the expression of Cyclin D1, a cell cycle regulator, and accelerate the progression of the cell cycle by inhibiting cycle-dependent kinase (CDK) inhibitor through AKT, known as protein kinase B, activation [43,44,45,46]. In addition, VEGF acts as an angiogenesis-promoting factor that induces the proliferation of vascular endothelial cells in association with Cyclin D1 [47,48]. Angiogenesis in the skin is directly related to nutritional supply and is a factor affecting the entire skin such as wound healing, metabolism and collagen synthesis [49,50,51,52,53,54]. Therefore, the increase in Cyclin D1 and growth factors by treatment with PPR-334 in NHDF suggests the possibility for dermato-physiological efficacy beyond the proliferation and anti-aging effect of dermal cells. Since this study did not examine vascular endothelial cells, further studies are needed to expand the scope by investigating the efficacy of PPR-334 in vascular endothelial cells or skin models containing endothelial components.

The efficacy of PPR-334 showed inconsistency across concentration. The results of MMP-1 gene expression showed a significant decrease even at 25 µg/mL of PPR-334 in vitro, while the results of the human artificial skin tissue models showed the opposite effect at a low concentration. However, in the case of CAT gene expression, the efficacy was reversed at 100 µg/mL PPR-334 in the human artificial skin tissue models. These findings suggest that PPR-334 may not be strictly concentration-dependent for all cases and determining an appropriate content and concentration of PPR-334 for research and development may have important implications.

## 4. Materials and Methods

### 4.1. Purification of PPR-334

The fermentation broth of *Saccharomyces cerevisiae*, supplied by CutisBio Co., Ltd. (Seoul, Republic of Korea), was used. The broth was centrifuged to separate the cell pellet and supernatant. From the supernatant obtained, a 10 L portion was stirred at ambient temperature (25 °C), followed by pH adjustment to 3.0 using an aqueous HCl solution. The acidified solution was filtered through a membrane with a pore size of ≤1 µm. The filtrate was then treated with 3 L of a non-polar polymeric adsorption resin (HP 20, DIAION, Samyang Corp., Seoul, Republic of Korea) under continuous stirring. After stirring, the mixture was filtered to remove the resin. The clarified filtrate was supplemented with 300 g of activated carbon (NORIT, Shanghai, China) and stirred for 30 min to allow adsorption of PPR-334. The activated carbon containing the adsorbed compound was collected by filtration and extracted with 5 L of methanol. The methanolic extract was subsequently concentrated under reduced pressure to obtain the PPR-334-enriched concentrate derived from the filtrate.

PPR-334 was purified from metabolic extracts of Saccharomyces cerevisiae using a CombiFlash RF system (Teledyne ISCO, Inc., Lincoln, NE, USA) equipped with a WELUX preparative reverse-phase C18 column (330 g, particle size 20–45 µm; Intertech Co., Ltd., Seoul, Republic of Korea). Elution was performed using distilled water adjusted to pH 3.0 with 1 M HCl at a flow rate of 60 mL/min. The eluent was monitored using a UV detector (Teledyne ISCO, Lincoln, NE, USA). set at 330 nm, and fractions containing PPR-334 were collected. These fractions were subsequently frozen at −70 °C for 24 h and lyophilized to yield a high-purity white powder of PPR-334 [55]. The structure of PPR-334 was confirmed by ^1^H NMR (500 MHz, D_2_O), and the spectral data are provided in the Supplementary Information.

### 4.2. 2,2-diphenyl-1-picrylhydrazyl (DPPH) Radicals Scavenging Assay

Given that DPPH radicals are stable free radicals, the antioxidant capacity of PPR-334 was assessed using a DPPH assay. Ascorbic acid (Samchun Chemical Co., Ltd., Seoul, Republic of Korea) was used as a positive control. A solution of 0.2 mM DPPH was prepared in 99% ethyl alcohol (Samchun Chemical Co.), and various concentrations of PPR-334 or ascorbic acid were mixed with DPPH solution and incubated for 10 min in the dark to prevent light-induced degradation. The absorbance was measured at 520 nm using a microplate reader (Biotek, Winooski, VT, USA).

### 4.3. 2,2′-Azino-bis(3-ethylbenzothiazoline-6-sulfonic Acid) Radical Cation (ABTS^+^•) Scavenging Assay

The total antioxidant activity of the sample was measured using an ABTS^+^• decolorization assay, following the method described by Roberta et al. [56]. Ascorbic acid was used as a positive control. The ABTS working solution was prepared by reacting 7.4 mM ABTS aqueous solution with 2.5 mM potassium persulfate in the dark for 16 h to generate ABTS^+^•. In total, 10 µL of various concentrations of PPR-334 or ascorbic acid and 190 µL of the ABTS^+^• working solution were mixed. Then, the mixture was incubated for 30 min in the dark. The absorbance was measured at 734 nm using a microplate reader.

### 4.4. Cell Cultures

NHDF (Promocell, Heidelberg, Germany) were cultured in Fibroblast Growth Medium (Promocell) containing supplement mix and 1% penicillin–streptomycin (PS; Gibco, Grand Island, NY, USA). HEKa (Gibco) were cultured in Epilife medium (ThermoFisher, Waltham, MA, USA) with Keratinocyte Growth Supplement (HKGS, Gibco) and 1% PS. All cells were grown at 37 °C in a humidified incubator (ThermoFisher) with 5% CO_2_.

### 4.5. Cell Cytotoxicity Assay

The cytotoxicity assay was assessed using the Quanti-Max^TM^ WST-8 Cell Viability Kit (Biomax, Guri-si, Gyeonggi-do, Republic of Korea). HEKa and NHDF seeded in 96-well culture plates were incubated for 24 h and then treated with PPR-334 at concentrations of 3.125–200 µg/mL. After 24 h of treatment, 10% WST-8 solution was added to each well and incubated for 1 h. The absorbance was measured at 450 nm using a microplate reader.

### 4.6. H_2_O_2_-Induced Cell Death Inhibition Assay

After 24 h for stabilization of HEKa and NHDF seeded in 96-well plates, the cells were treated with various concentrations of PPR-334 and H_2_O_2_ (Sigma-Aldrich, St. Louis, MO, USA), a reactive oxygen species used to induce cell death, for an additional 24 h. Then, 10% WST-8 solution was added to each well and incubated for 1 h. The absorbance was measured at 450 nm using a microplate reader.

### 4.7. 2′,7′-Dichlorofluorescin Diacetate (DCF-DA) Assay

HEKa (2.5 × 10^4^) were seeded in 96-well black plates and stabilized for 24 h. Then, the cells treated with the indicated concentrations of PPR-334 or ascorbic acid for 3 h. Ascorbic acid was used as a positive control. All solvents or washing solutions used for the assay were diluted from 10× HBSS (Welgene, Gyeongsan-si, Republic of Korea) to 1X HBSS. H2DCFDA (Invitrogen, Carlsbad, CA, USA) was diluted to 50 mM in dimethyl sulfoxide (DMSO; Sigma-Aldrich) and used to treated cells for 30 min. After the treatment, UVB was irradiated at an intensity of 20 mJ/cm^2^ and then incubated for 2 h.

### 4.8. UVA/B Irradiation

HEKa (4.5 × 10^5^) were cultured in 6-well plates for 24 h. Cells were irradiated with UVB (15 mJ/cm^2^) or UVA (2 J/cm^2^) using Vilber UV Bio-sun (Vilber Lourmat, Marne-la-Vallée, France). During irradiation, cells were maintained in PBS either with or without various concentrations of PPR-334, corresponding to the treatment and control group, respectively.

### 4.9. Quantitative Reverse Transcription PCR (qRT-PCR) Assay

HEKa (4.5 × 10^5^) and NHDF (4 × 10^5^) were seeded in 6-well plates or 35 mm dishes. Human artificial skin tissue models were mechanically disrupted using scissors. RNA was extracted using TRIzol reagent (Invitrogen). cDNA was synthesized from total RNA using the GoScript Reverse Transcription System (Promega, Madison, WI, USA) and T100^TM^ Thermal Cycler (Biorad, Hercules, CA USA). qPCR was carried out using SYBR Green Supermix (Biorad) and specific primer obtained from Biorad and the gene expressions were measured using CFX Connect^TM^ Real-Time System (Biorad). mRNA levels of glyceraldehyde-3-phosphate dehydrogenase (GAPDH) were used for normalizing all results.

### 4.10. Cell Proliferation Assay

To visually monitor cell proliferation, NHDF (2 × 10^4^) were seeded in 6-well plate and stabilized for 24 h. Cells were serum-starved for 12 h, treated with 25 µg/mL PPR-334 and monitored using live-cell imaging system (JuLI^TM^ Stage, NanoEntek, Seoul, Republic of Korea) for up to 72 h. For quantitative assessment of proliferation, NHDF (3 × 10^3^) and HEKa (5 × 10^3^) were cultured in 96-well plate and treated with 12.5–50 µg/mL PPR-334 for 72 h. Cells were then treated with 10% WST-8 solution for 2 h. The absorbance at 450 nm was measured using a microplate reader.

### 4.11. Immunocytochemistry (ICC) Assay

NHDF (1.3 × 10^4^) or HEKa (2 × 10^4^) were cultured in 8-well culture slides for 24 h. Cells treated with 25 µg/mL PPR-334 and then washed twice with 1× phosphate-buffered saline (PBS; Biosesang, Seoul, republic of Korea). Cells were fixed with 4% paraformaldehyde solution (Bylabs, Hanam-si, Gyeonggi-do, Republic of Korea) for 30 min and permeabilized with 0.1% Triton^TM^ X-100 (Sigma-Aldrich) for 15 min. Cells were blocked for 1 h with PBS containing 5% bovine serum albumin (BSA; Bioworld Biotech Co., Ltd., Nanjing, China) and incubated overnight at 4 °C with anti-collagen I antibody (1:2500; ab138492; Abcam, Cambridge, UK) and NRF2 (1:200; #12721; Cell Signaling Technology, Danvers, MA, USA). Cells were then washed three times with PBS containing 0.5% BSA and incubated with Alexa Fluor 488-conjugated anti-rabbit IgG secondary antibody (1:1000; #4412S, Cell Signaling Technology), or Alexa Fluor 594-conjugated anti-rabbit IgG secondary antibody (1:1000; #8889S, Cell Signaling Technology), for 1 h at room temperature. Nuclei were counterstained with DAPI using a mounting medium containing DAPI (ab104139, Abcam). Cells were performed using fluorescence scope.

### 4.12. Anti-Glycation Assay

A cell-free glycation assay was conducted using BSA, 2.5% dissolved in PBS. A reaction mixture consisting of 2.5% BSA, 1 M glucose, and PPR-334 at different concentrations was prepared at a ratio of 9.5:9.5:1 in 96-well black plate. Aminoguanidine (AG) was included as a positive control. The plate was then sealed with a cover film and incubated at 50 °C for 7 days. The formation of advanced glycation end-products (AGEs) was quantified by measuring autofluorescence at an excitation wavelength of 340 ± 40 nm and an emission wavelength of 440 ± 40 nm using a microplate reader, with aminoguanidine serving as the positive control.

### 4.13. Label-Free Collagen Imaging

A full-thickness human skin equivalent (Epi-DermFT^TM^, #EFT-400, MatTek, Ashland, MA, USA) and the culture medium (#EFT-400-ASY) were purchased from MatTek corp. The skin equivalents were irradiated with UVA (5 J/cm^2^) and PPR-334 was treated on the surface of the skin equivalent. Then, the skin equivalents were incubated in a 37 °C, 5% CO_2_ incubator for 48 h and fixed with 4% formaldehyde. The experiment was performed in quadruplicate. Collagen fibers in the skin equivalents were three-dimensionally visualized by a multiphoton microscopy (#LSM980 NLO, Carl Zeiss AG, Oberkochen, Germany) using a second harmonic generation technique. Total measurement volume is 300(x) × 300(y) × 70(z) μm^3^. The volumetric signal intensities of collagen fibers were analyzed by Image-Pro Premier 3D v9.1 software (Media Cybernetics Inc., Bethesda, MD, USA).

### 4.14. Western Blot Analysis

NHDF (4 × 10^5^) were seeded in 35 mm dishes and treated with PPR-334 (25 µg/mL) for 1–24 h. HEKa (4.5 × 10^5^) were seeded in 35 mm dishes and treated with UVB 30 mJ/cm^2^ (or not) and PPR-334 (25 µg/mL) for 1–12 h. Cells were washed twice with 1X PBS, and total intracellular proteins were extracted using 1× radioimmunoprecipitation assay buffer (RIPA; Cell Signaling Technology) containing protease inhibitor and phosphatase inhibitor (Millipore, Merk KGaA, Darmstadt, Germany). The protein content was quantified by Pierce BCA Protein Assay Kit (ThermoFisher). Quantified proteins were separated by sodium dodecyl sulfate-polyacrylamide gel electrophoresis (SDS-PAGE) and transferred to nitrocellulose membrane (Biorad). The membranes were blocked for 2 h with 1× Tris-buffered saline (TBS; Biorad) containing 5% Blotting-Grade Blocker (Biorad) and incubated overnight at 4 °C with primary antibodies against c-Myc (1:2000; 10828-1-AP; Proteintech Group, Inc., Rosemont, IL, USA), Cyclin D1 (1:1000; 2978S; Cell Signaling Technology), Ki-67 (1:500; ab16667; Abcam), Bax (1:5000; ab32503; Abcam), Caspase-9 (1:1000; 20750S; Cell Signaling Technology), NRF2 (1:1000; #12721; Cell Signaling Technology) and β-actin (1:1000; ab179467; Abcam). The following day, membranes were washed three times with 1× TBS containing 0.05% Tween-20 (Samchun) and incubated with secondary antibody (ab6728; Abcam), diluted 1:10,000, for 1 at room temperature. Immunoreactive proteins were visualized using Clarity Max Western ECL Substrate (Biorad) and SuperSignal^TM^ West Femto Maximum Sensitivity Substrate (ThermoFisher) and imaged using an iBright CL750 Imaging System (Invitrogen).

### 4.15. Statistical Analysis

The data was expressed as the mean ± standard deviation (SD) from at least three independent experiments (*n* = 3), and the statistical significance was determined using Student’s *t*-test for comparisons between two groups. For comparisons involving three or more groups, one-way ANOVA followed by Tukey’s post hoc was applied. A *p*-value < 0.05 was considered statistically significant.

## 5. Conclusions

In this study, we conducted the experimental validation using Porphyra-334 (PPR-334) that was biosynthesized and purified from engineered *Saccharomyces cerevisiae* through a synthetic biology approach. By utilizing a single, high-purity compound produced at scale, we addressed the limitations of low natural abundance and inefficient extraction from marine sources, thereby enhancing the feasibility of industrial application. The biological efficacy of PPR-334 was confirmed in both primary cells and human artificial skin tissue models, where it demonstrated significant antioxidant and anti-aging effects, even when applied alone. These results support the potential of PPR-334 not only as a safe and effective cosmetic ingredient but also as a multifunctional material with dual or multiple benefits. Overall, our findings suggest that this integrated biosynthesis and purification strategy provides a sustainable and scalable alternative to traditional methods, paving the way for the broader use of high-purity MAAs in cosmetic formulations.

## Data Availability

Data supporting the findings of this study are available from the corresponding author on request.

## Supplementary Materials

The following supporting information can be downloaded at: https://www.mdpi.com/article/10.3390/md24030098/s1, Supplementary Information: Chemical structure and ^1^H NMR data (500 MHz, Water-d2) of PPR-334; Table S1: Radical activity inhibition rate; Figure S1: Cytotoxicity of PPR-334 in HEKa; Table S2: DCF-DA fluorescence inhibition rate; Figure S2: Cytotoxicity of PPR-334 in NHDF. Figure S3: H_2_O_2_-induced cell death inhibition assay in NHDF. NHDF were co-treated H_2_O_2_ and PPR-334 for 24 h. Ascorbic acid served as positive control. The cell viability was measured by WST-8 assay. Results are presented as mean ± SD. ### *p* < 0.001 vs. control group. ** *p* < 0.01, *** *p* < 0.001 vs. H_2_O_2_ alone. Figure S4: CAT mRNA expression in NHDF treated PPR-334. NHDF were treated with PPR-334 for 24 h. The gene expression level of CAT was measured by qRT-PCR. Results are presented as mean ± SD. ** *p* < 0.01 vs. control group. Figure S5: Proliferation effect of PPR-334 in HEKa. HEKa were treated with PPR-334 for 72 h. The proliferation rate was determined by WST-8 assay. Results are presented as mean ± SD. *** *p* < 0.001 vs. control group.

## Abbreviations

The following abbreviations are used in this manuscript:

ABTS	2,2′-azino-bis(3-ethylbenzothiazoline-6-sulfonic acid)
AG	Aminoguanidine
AGEs	Advanced glycation end-products
Bax	Bcl-2-associated X protein
Bcl-2	B-cell lymphoma 2
BSA	Bovine serum albumin
CAT	Catalase
CDK	Cycle-dependent kinase
c-Myc	Cellular myelocytomatosis oncogene
COL1A1	Collagen type I alpha 1
Cyclin D1	G1/S-specific cyclin D1
DCF-DA	2′,7′-dichlorofluorescin diacetate
DMSO	Dimethyl sulfoxide
DPPH	2,2-diphenyl-1-picrylhydrazyl
EGF	Epidermal growth factor
ECM	Extracellular matrix
GAPDH	Glyceraldehyde-3-phosphate dehydrogenase
GPX4	Glutathione peroxidase 4
HEKa	Human epidermal keratinocyte cells
HRP	Horseradish peroxidase
ICC	Immunocytochemistry
IGF-1	Insulin-like growth factor-1
Keap1	Kelch-like ECH-associated protein 1
Ki67	Marker of proliferation Ki-67
MAAs	Mycosporine-like amino acid
MMP-1	Matrix metalloproteinase-1
NF-_K_B	Nuclear factor kappa B
NHDF	Normal human dermal fibroblast cells
NRF2	Nuclear factor erythroid 2-related factor 2
PBS	Phosphate-buffered saline
PPR-334	Porphyra-334
qRT-PCR	Quantitative reverse transcription PCR
RIPA	Radioimmunoprecipitation assay buffer
ROS	Reactive oxygen species
SD	Standard deviation
SDS-PAGE	Sodium dodecyl sulfate-polyacrylamide gel electrophoresis
SOD	Superoxide dismutase
TBS	Tris-buffered saline
TIMPs	Tissue inhibitors of metalloproteinase
UV	Ultraviolet
VEGF	Vascular endothelial growth factor
