# Supplementary material for: Antioxidant and Anti-Aging Effects of Porphyra-334 Produced from Saccharomyces cerevisiae in Human Skin Models
Source: Mar Drugs. 2026 Feb 28;24(3):98. doi: 10.3390/md24030098 (PMC13027577; doi:10.3390/md24030098)
Supplement: Supplementary file 1 [file marinedrugs-24-00098-s001.zip › marinedrugs-4020210-supplementary.pdf]

Supplementary Information

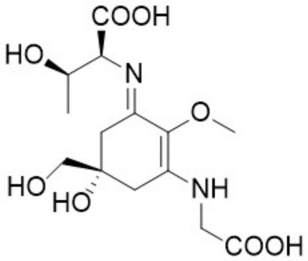

Chemical structure and ^{1}H NMR data (500 MHz, Water-d_2) of PPR-334. δH 4.44 (m, H-2), 4.36 (d, *J* = 3.8 Hz, H-3), 4.25 (d, *J* = 3.4 Hz, H-13), 3.69 (s, H-11), 3.58 (s, H-12), 2.90 (d, *J* = 17.4 Hz, H-10), 2.86 (d, *J* = 17.4 Hz, H-8), 2.78 (d, *J* = 17.4 Hz, H-8), 2.76 (d, *J* = 17.5 Hz, H-10), 1.25 (d, *J* = 6.6 Hz, H-1).

Supplementary Figure

Table S1. Radical activity inhibition rate

Radical activity inhibition rate			
Compound	Conc.	DPPH radical inhibition rate	ABTS radical inhibition rate
Ascorbic acid (ppm)	5	40 ± 0.49	25 ± 0.08
	10	63 ± 0.7	52 ± 0.71
	20	≥95	≥95
PPR-334 (μg/mL)	12.5	40 ± 1.87	31 ± 0.52
	25	38 ± 7.37	41 ± 0.17
	50	38 ± 3.61	46 ± 0.25

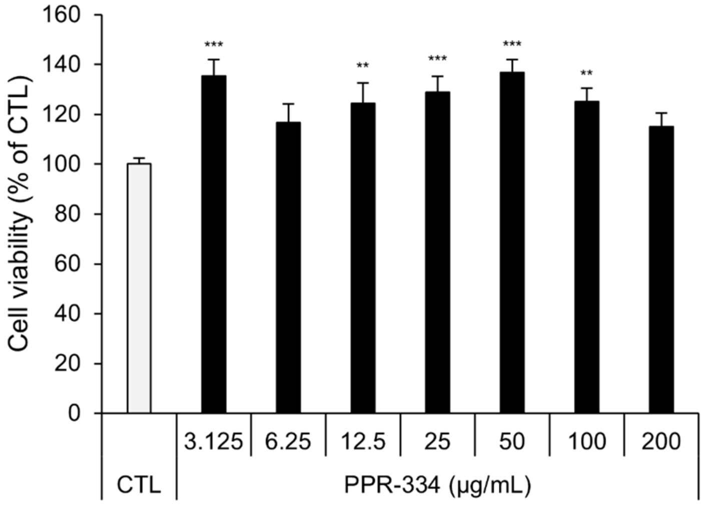

Figure S1. Cytotoxicity of PPR-334 in HEKa cells. HEKa were treated with various concentration of PPR-334 (3.125 – 200 μg/mL) for 24 h. The cell viability was determined

using WST-8 assay. Results are presented as mean ± SD. ** $p < 0.01$, *** $p < 0.001$ vs. control group.

Table S2. DCF-DA fluorescence inhibition rate

ROS inhibition rate		
Compound	Conc.	Inhibition rate (%)
Ascorbic acid (ppm)	50	61 ± 6.19
	100	93 ± 6.19
	200	≥ 95
PPR-334 (μg/mL)	12.5	86 ± 6.19
	25	79
	50	79

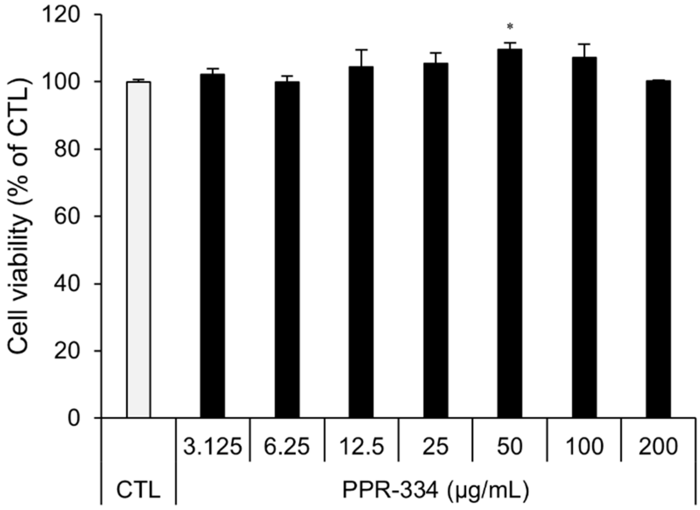

Figure S2. Cytotoxicity of PPR-334 in NHDF cells. NHDF were treated with various concentrations of PPR-334 (3.125 – 200 μg/mL) for 24 h. The cell viability was measured by WST-8 assay. Results are presented as mean ± SD. * $p < 0.05$ vs. control group.

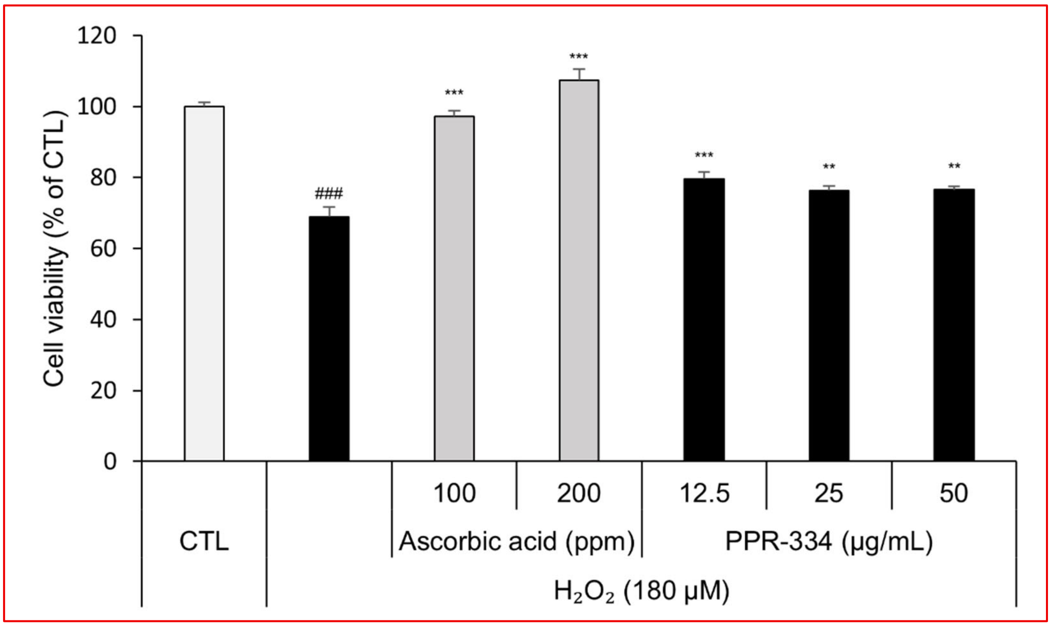

Figure S3. H_2O_2-induced cell death inhibition assay in NHDF. NHDF were co-treated H_2O_2 and PPR-334 for 24 h. Ascorbic acid served as positive control. The cell viability was measured by WST-8 assay. Results are presented as mean ± SD. ### $p < 0.001$ vs. control group. ** $p < 0.01$, *** $p < 0.001$ vs. H_2O_2 alone.

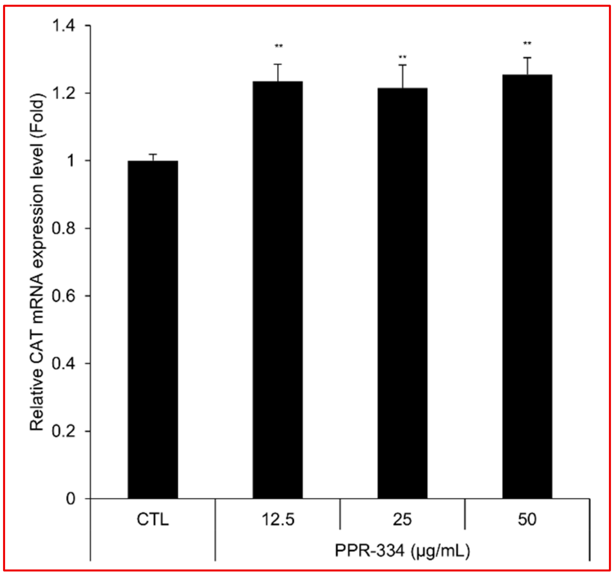

Figure S4. CAT mRNA expression in NHDF treated PPR-334. NHDF were treated with PPR-334 for 24 h. The gene expression level of CAT was measured by qRT-PCR. Results are presented as mean ± SD. ** $p < 0.01$ vs. control group.

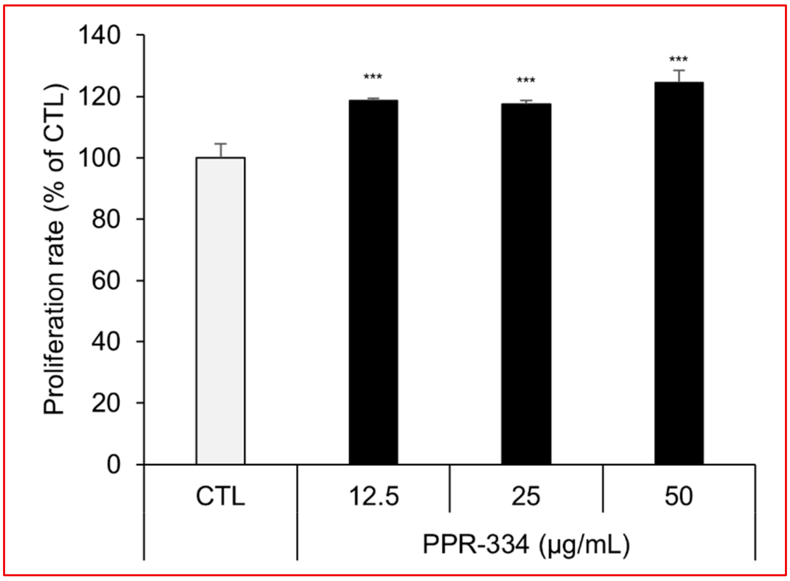

Figure S5. Proliferation effect of PPR-334 in HEKa. HEKa were treated with PPR-334 for 72 h. The proliferation rate was determined by WST-8 assay. Results are presented as mean ± SD. *** $p < 0.001$ vs. control group.
